# Molybdenum Oxide Thin Films Grown on Flexible ITO-Coated PET Substrates

**DOI:** 10.3390/ma14040821

**Published:** 2021-02-09

**Authors:** Alice Marciel, Manuel Graça, Alexandre Bastos, Luiz Pereira, Jakka Suresh Kumar, Joel Borges, Filipe Vaz, Marco Peres, Sergio Magalhães, Katharina Lorenz, Rui Silva

**Affiliations:** 1 Institute for Nanostructures, Nanomodelling and Nanofabrication (i3N), Physics Department, University of Aveiro, Campus de Santiago, 3810-193 Aveiro, Portugal; alicemarciel@ua.pt (A.M.); luiz@ua.pt (L.P.); suresh@ua.pt (J.S.K.); 2 Centre for Research in Ceramics and Composite Materials (CICECO), Aveiro Institute of Materials, Department of Materials and Ceramic Engineering, University of Aveiro, 3810-193 Aveiro, Portugal; acbastos@ua.pt (A.B.); rsilva@ua.pt (R.S.); 3 Centro de Física, Universidade do Minho, 4710-057 Braga, Portugal; joelborges@fisica.uminho.pt (J.B.); fvaz@fisica.uminho.pt (F.V.); 4 Instituto de Plasmas e Fusão Nuclear (PFN), Instituto Superior Técnico (IST), Campus Tecnológico e Nuclear, Estrada Nacional 10, 2695-066 Bobadela LRS, Portugal; marcoperes@ctn.tecnico.ulisboa.pt (M.P.); smagalhaes@ctn.tecnico.ulisboa.pt (S.M.); lorenz@ctn.tecnico.ulisboa.pt (K.L.); 5 Instituto de Engenharia de Sistemas de Computadores-Microsystems and Nanotechnology (INESC-MN), IST, 1000-029 Lisboa, Portugal

**Keywords:** MoO_x_, flexible substrates, electrochromic, DC magnetron sputtering

## Abstract

Molybdenum oxide thin films were deposited on stiff and flexible substrates by reactive DC magnetron sputtering. Two sets of samples were prepared. The first with different O_2_/Ar flow rate ratios and the second, fixing the oxygen content, with different time of deposition. As the O_2_/Ar flow rate ratio varies from 0 up to 0.56, a threshold was found, ranging from crystalline to amorphous nature, and from a nontransparent appearance with metallic-like electrical conductivity to transparent and dielectric behaviour. From the second set, all transparent, the MoO_x_ films present a compact/dense and featureless morphology with thickness from 190 up to 910 nm, depending on the time of deposition. Their structure was corroborated by XPS and Rutherford Backscattering Spectrometry (RBS) and density measurements were performed by RBS and X-ray reflectivity (XRR), revealing a value of 2.4 g/cm^3^. The surface roughness is in the order of a few nanometers and the maxima optical transmission, in the visible range, is around 89%. Electrochemical cyclic voltammograms showed noticeable color reversibility and reproducibility on the flexible substrates opening new framework possibilities for new electrochomic devices.

## 1. Introduction

Thin films based on oxide materials have gained momentum in recent years due to their properties that include high optical transparency, good electrical carrier mobility, and mechanical stress tolerance. The possibility of tailoring their physical properties, resulting in different electronic and geometric structures that can exhibit metallic, semiconducting, or insulating characters, makes this class of materials unique. This increases their importance as potential materials in a wide range of applications that include transparent electronics, optoelectronics, magneto electronics, photonics, spintronics, thermoelectrics, piezoelectrics, power harvesting, hydrogen storage and environmental waste management [1,2].

Beyond all amazing properties related to oxide materials, there is a well-known group of metal oxides (from Ti, V, Cr, Mn, Fe, Co, Ni, Nb, Mo, Rh, Ta, W and Ir) that can exhibit electrochromic behaviour, reversely changing their optical characteristics in a persistent mode under the action of an electrical field. The optical changes quantified by transmittance and/or reflectance are associated with an electrochemically induced oxidation-reduction reaction [3,4,5,6].

The electrochromic behaviour of molybdenum oxide (MoO_3_) was discovered around 1980 [7]. This oxide, in parallel with tungsten oxide (WO_3_), is one of the most studied electrochromic materials [3,5]. Several techniques of deposition have been used to grow MoO_3_ films such as sputtering [8,9,10], thermal evaporation [11], sol-gel [12,13], electron beam evaporation [13,14], chemical vapour deposition [15,16], pulsed laser deposition [17,18], spray pyrolysis [19,20] and atomic layer deposition [21,22]. However, the electrochromic performance of a device based on these films is still a big scientific and technological challenge. The electrochromic performance depends on multiple factors, for example: deposition technology used to produce thin films with controlled porosity; type of microstructure; stoichiometry; combination of high optical transparency with high electrical conductivity of the transparent conducting oxide (TCO) used; adhesion of the thin film layers in the sandwich approach; high chemical stability; management of the process of charge insertion/extraction and charge balancing; and electrolytes quality when in contact with the electrolyte environment [23,24,25,26].

The improvement of the electrochromic performance and properties is being investigated by mixing some of the simple metal oxides, as MoO_3_, WO_3_ or V_2_O_5_, at the nanometric scale, forming new materials with coloring tuning, low response time, higher contrast ratio, etc. [4,27,28,29,30]. It is known that many cycles between coloring and bleaching tend to degrade the electrochromic performance and some efforts to study the rejuvenation of MoO_3_ thin films are being done [7,31]. Despite the electrochromic characteristic, MoO_3_ films are also interesting materials for energy applications [3].

In this work, MoO_x_ thin films were grown by reactive DC magnetron sputtering, in function of O_2_/Ar flow rate ratio and deposition time, onto different substrates that include polyethylene terephthalate (PET) and glass, both with indium tin oxide (ITO) conductive layer, and glass plates without ITO and silicon. The structural, chemical, optical, electrical, and electrochemical properties were studied.

## 2. Materials and Methods

### 2.1. Thin Films Preparation

MoO_x_ thin films were grown by reactive DC magnetron sputtering, using a 60 L custom-made laboratory sized deposition system [32]. MoO_x_ thin films were obtained using a 200 × 100 × 6 mm^3^ metallic Mo target with 99.95% of purity, sputtered by applying a current density of 100 A/m^2^ in a plasma composed of argon (Ar, working gas), and a reactive gas that was oxygen (O_2_). The target was placed at 7 cm from the rotating substrate holder. Before the film deposition, the sputtering system was evacuated to 1 × 10^−3^ Pa. Argon and oxygen, both of 99.997% of purity, were introduced through mass flow-controlled gas inlets.

For the first series of samples, different O_2_/Ar flow rate ratios were used to prepare a set of films with different Mo oxidation states. The flow of Ar was kept constant in all depositions (25 sccm, corresponding to a partial pressure of 3.9 × 10^−1^ Pa) and only the O_2_ flow was changed. The O_2_/Ar flow ratios used were 0, 0.16, 0.32, 0.48 and 0.56. The time of deposition was kept for 60 min in all of these samples. In the second series, the O_2_/Ar gas flow ratio was fixed at 0.56 and the deposition time was decreased from 60 min (used in the first series) to 30 min and to 15 min, to prepare MoO_x_ with different thicknesses. No external heating was used during the deposition and the substrates were grounded.

All substrates were previously cleaned by plasma treatment using a Zepto plasma system (Diener Electronic, Ebhausen, Germany), first with oxygen for 5 min and then with argon for 15 min. It must be noted that the polyethylene terephthalate (PET) and glass substrates, both with an indium tin oxide (ITO) conductive layer, present a sheet resistance of 5 Ω/▯ and 60 Ω/▯, respectively. The p-type silicon substrate (Boron doped) with (100) orientation, shows a resistivity from 5–10 Ω × cm.

### 2.2. Characterization

#### 2.2.1. Microstructural and Morphologic Characterization

The as-deposited films were undergone for characterization. Their structure was analyzed by an X-ray diffractometer (PANalytical XPert-Pro, Almelo, The Netherlands) with CuKα radiation (λ = 1.54060 nm), operating at a scan rate of 0.01°/s over a 2θ range of 10–80°. The crystallite size was calculated using the Scherrer equation. X-ray reflectivity (XRR) measurements were performed on a Bruker D8 AXS diffractometer equipped with a Cu source and a soller slit in the secondary beam path to decrease the horizontal divergence. A 0.2 mm width slit and a Ni filter were placed at the primary beam side to reduce the horizontal beam divergence and to eliminate the K-beta line, respectively. Atomic compositions and the depth concentration profile (in the growth direction) were measured by Rutherford Backscattering Spectrometry (RBS), with a 2 MeV α particle beam of 1 mm diameter obtained from a Van de Graaff accelerator. The random spectra were obtained by tilting the sample by 5 degrees and rotating the sample during the measurement to avoid channeling in the substrate. The backscattered particles were detected using two PIN diode detectors mounted at different backscattering angles of 165° and 140°. The compositions were extracted as a function of depth by a fitting procedure, using the nuclear data furnace code (NDF) [33]. XPS spectra were performed with a Kratos Axis Ultra HAS spectrometer using the Al Kα excitation line at 1487 eV. XPS signals were calibrated using the C1s peak at 285 eV resulting from the accidental hydrocarbons layer present on the sample surface. For these measurements, an energy step size of 0.1 eV and and a pass energy of 40 eV were used, while the analyzed area was 300 µm × 700 µm. Raman spectra were obtained with a Horiba Jobin Yvon HR800 spectrometer using the 532 nm line, of a 50 mW He-Cd laser. Surface, cross-section morphology and thin-film thickness estimation were carried out by scanning electron microscopy (SEM) using a Hitachi SU-70 and surface roughness was evaluated using an S-NEOX 3D Optical Profiler system (Sensofar Metrology, Terrassa, Spain).

#### 2.2.2. Optical Characterization

For the optical characterization, the transmittance spectra under normal incidence in the visible range were recorded using a Shimazu UV-2100 spectrometer (Shimadzu Corporation, Kyoto, Japan).

#### 2.2.3. Electrical Characterization

DC and AC electrical measurements were performed at room temperature. The resistivity of the bulk MoO_x_ thin films grown on a silicon substrate at 0, 0.16, 0.32 and 0.48 O_2_/Ar flow rate ratio resulted from the measurement of I-V characteristics, and the apparatus used was a multimeter 34401A (HP) and a laboratory power supply ISO-Tech Dual Tracking with 5 V fixed model IPS 2303 DD (Isotech, Conchester, VT, USA). The thin film grown on PET-ITO substrate at 0.56 O_2_/Ar flow rate ratio was also analyzed by impedance spectroscopy (Agilent 4294A Precision Analyser, Santa Clara, CA, USA). For that, a matrix of gold electrode pads of 2 mm in diameter, separated by 2 mm, were deposited on the thin film surface using a sputter coater SC7620 (Quorum, East Sussex, UK). The connection between the sample and the instrument cell was made using micropositioners with gold probes.

#### 2.2.4. Electrochromic Characterization

Cyclic voltammetry experiments were performed inside a glove box with an argon-controlled atmosphere, using a Methrom Autolab PGstat 302N, in a 3-electrode arrangement, with the samples as “working electrodes”, a platinum wire as counter-electrode and a low leakage Ag/AgCl/KCl (3 M) as reference electrode. The voltammograms were swept from −2.5 to +2.0 V_Ag/AgCl/KCl (3 M)_. In situ and simultaneously with the cyclic voltammetry, optical transmittance was recorded using an Ocean Optics USB4000 spectrometer (Ocean Optics Inc., Dunedin, FL, USA).

## 3. Results and Discussion

### 3.1. Microstructural and Morphologic Characterization

#### 3.1.1. Structural Analysis

**Figure 1:**
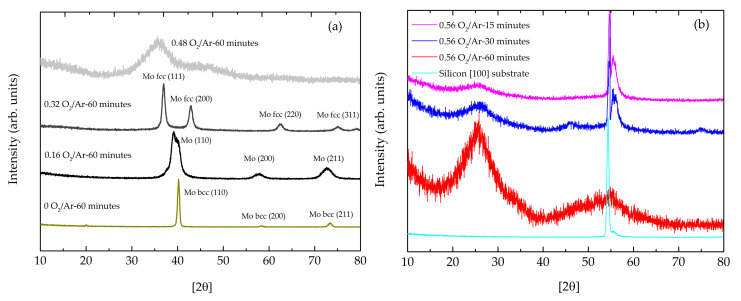
X-ray diffraction patterns of: (**a**) the first series of MoO_x_ thin films prepared with different gas flow rate ratio of O_2_/Ar (all samples of the first series have metallic-like appearance); (**b**) the second series of MoO_x_ thin films prepared with the same gas flow rate ratio of O_2_/Ar and different times of deposition (all samples have transparent appearance).

Figure 1a presents the XRD patterns of the first series of MoO_x_ films deposited onto silicon substrates. All films deposited at a gas flow rate ratio of O_2_/Ar less or equal to 0.32 are polycrystalline. The films deposited at flow rate ratios less or equal to 0.16 O_2_/Ar have body-centered cubic (bcc) molybdenum structure matching with JCPDS Card No. 04-002-0890 [34,35,36]. Films deposited at a flow rate ratio of 0.32 O_2_/Ar show face-centered cubic (fcc) molybdenum structure with correspondence with JCPDS Card No. 01-0882331. This transition, from bcc to fcc, can be related to strains induced by the oxygen accommodation on the crystal lattice [37] and it was responsible for the diffraction peak shift to lower angles. The calculated crystallite sizes (Table 1) showed a decrease from 28 nm to 13 nm with the increase of O_2_/Ar flow ratio.

The increase of the oxygen content caused a broadening of the diffraction peaks. The sample deposited with the flow rate ratio of 0.48 O_2_/Ar showed a broadened band, centered at 2θ ≈ 35°, which suggested a coexistence between an amorphous structure and some few nanocrystallites dispensed in the matrix. At the flow rate ratio of 0.56 O_2_/Ar, corresponding to the second series of depositions, the diffractogram pattern showed in Figure 1b, with broadband centered at 2θ ≈ 25°, confirmed the formation of an amorphous structure based on the MoOx units [38,39,40]. In the samples grown in silicon, the intensity of the peak corresponding to silicon was observed only on samples with sputtering time below 60 min, which inferred a lower sample thickness.

#### 3.1.2. Composition of the Films

**Figure 2:**
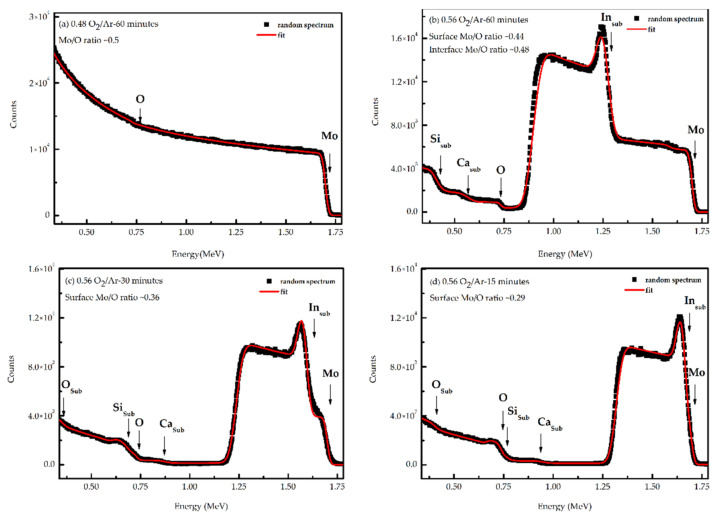
Rutherford Backscattering Spectrometry (RBS) spectrum obtained for measurements of the last sample from the first series: (**a**) deposited with a flow rate ratio of 0.48 O_2_/Ar for 60 min; and of the samples of the second series, prepared with the same flow rate ratio of 0.56 O_2_/Ar and different deposition times, (**b**) 60 min, (**c**) 30 min and (**d**) 15 min. The vertical arrows correspond to the surface/interface scattering energies of the different elements assigned to the film/substrate (sub).

Figure 2a–d show the RBS spectra of the MoO_x_ thin films, deposited onto the glass-ITO substrate in the transition region (from metallic to transparent like appearance) and in transparent condition for different times of deposition. The RBS spectrum of the sample grown at the flow rate ratio of 0.48 O_2_/Ar was characterized by two barriers assigned to the Mo and O and no barriers assigned to the substrate were present (Figure 2a). The absence of RBS signal from the substrate is due to the high thickness of the film, which should be higher than 1250 nm, a value estimated using the NDF code (considering 6.5 g/cm^3^ the volumetric density of MoO_2_ [41]). A good fit was obtained assuming a homogenous composition profile in depth with a Mo/O ratio of ~0.5. However, it should be noticed that the sensitivity of RBS to O was strongly reduced due to its low mass and to the overlap of the O-signal with the Mo signal. The spectra obtained from the samples grown at 0.56 flow rate ratio of O_2_/Ar, showed the end of the MoO_x_ layer meaning that these films were thinner, which is in agreement with XRD results that showed the appearence of the substrate (Si) peak. Furthermore, three extra barriers assigned to indium (In), calcium (Ca) and silicon (Si) from the substrate are marked in Figure 2b–d. To estimate the composition and the depth concentration profiles of Mo, these spectra were fitted using the NDF code (red lines in Figure 2). Regarding the film grown at the flow rate ratio of 0.56 O_2_/Ar for 60 min, the fit suggested a compositional gradient with an increase of the molybdenum concentration with depth. The Mo/O ratio as a function of the depth, estimated by the fit, changed from ~0.44 at the surface to ~0.48 at the interface with the substrate. The depth profile analysis, which was also a film thickness measurement, indicated that for an equal deposition time, the increase of O_2_/Ar ratio promoted a thinner film. The sample with a 0.48 O_2_/Ar ratio had a thickness higher than 1250 nm. This thickness decreased to approximately 450 nm for the sample with 0.56 O_2_/Ar ratio. For an equal O_2_/Ar ratio (0.56) and decreasing the sputtering time, the thickness decreased until around 200 nm for the sample growth with 30 min and 100 nm for the sample growth with 15 min. Another observation, for the samples grown at 30 and 15 min, is that the molybdenum profile was not homogeneous across the thickness. Both samples showed a maximum of molybdenum around half of the film thickness.

#### 3.1.3. Surface Chemistry Analysis

**Figure 3:**
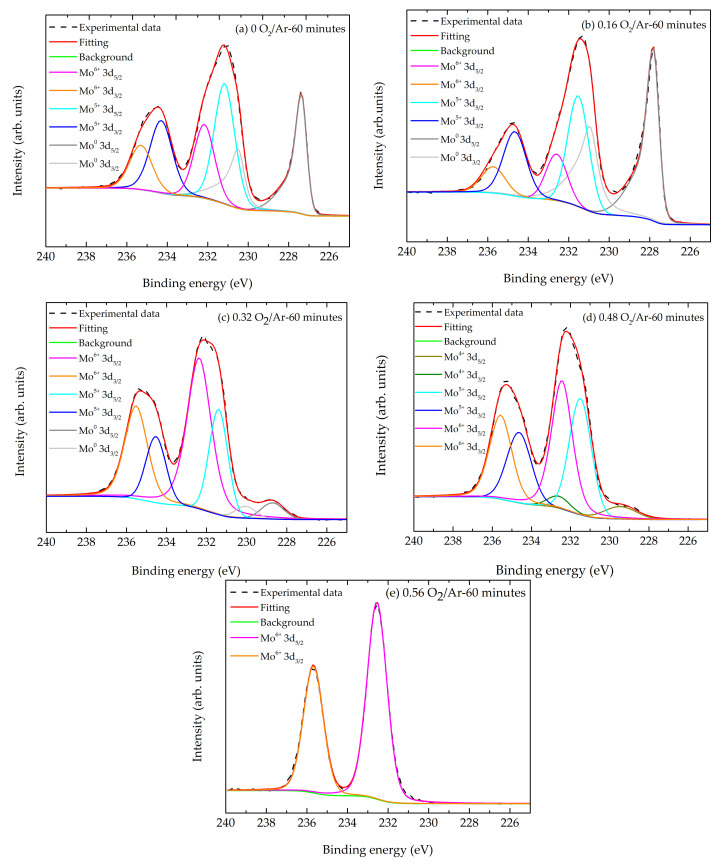
High-resolution XPS spectra of the Mo 3d sate showing the spin-orbit splitting. The deconvoluted peaks associated with the 3d_3/2_ and 3d_5/2_ orbitals are also indicated for sample (**a**) 0 O_2_/Ar-60 min, (**b**) sample 0.16 O_2_/Ar-60 min, (**c**) sample 0.32 O_2_/Ar-60 min, (**d**) sample 0.48 O_2_/Ar-60 min, (**e**) sample 0.56 O_2_/Ar-60 min.

The thin film’s surface chemistry was analyzed by X-ray photoelectron spectroscopy. Exhibited in Figure 3 is the high-resolution XPS spectrum of the Mo 3d state for samples grown at different O_2_/Ar ratios. The different spectra from (a) to (e) showed the spin-orbit splitting characteristic of the Mo 3d core level. This spectrum was deconvoluted into two doublets, each of them related to a specific oxidation state. Fitting with Lorentzian–Gaussian line shapes and assuming a spin-orbit splitting of 3.13 eV and an intensity ratio between 3d_5/2_ and 3d_3/2_ of 1.5, revealed the presence of Mo^6+^, Mo^5+^ and Mo^0^ oxidation states in the sample prepared without the introduction of oxygen (Figure 3a). The presence of Mo^6+^ and Mo^5+^ oxidation states might come from surface oxidation phenomena once the as-deposited films were exposed to atmospheric conditions. Figure 3b shows the core level XPS spectrum for the film deposited at a flow rate ratio of O_2_/Ar of 0.16, revealing that the surface composition of was similar to the previous. For the Mo^0^ state, the Mo 3d_5/2_ and Mo 3d_3/2_ positions were found at 227.37 eV and 230.50 eV (Figure 3a) and at 227.83 eV and 230.96 eV (Figure 3b), respectively for 0 and 0.16 O_2_/Ar ratios. The Mo^5+^ and Mo^6+^ oxidation states had Mo 3d_5/2_ and Mo 3d_3/2_ peak positions at 231.17 eV and 234.30 eV, and 232.17 eV and 235.30 eV (Figure 3a) and at 231.53 eV and 234.66 eV and 232.60 eV and 235.73 eV (Figure 3b), correspondingly. All these values were in agreement with the literature [42,43,44,45,46].

Figure 3c shows the core level XPS spectrum for representative thin films deposited at a flow rate ratio of O_2_/Ar of 0.32. For the Mo^0^ state the Mo 3d_5/2_ and Mo 3d_3/2_ positions were found at 228.71 eV and 230.96 eV and the Mo^5+^ and Mo^6+^ oxidation states had Mo 3d_5/2_ and Mo 3d_3/2_ peak positions at 231.40 eV and 234.53 eV and 232.37 eV and 235.52 eV, respectively. On Figure 3d, corresponding to the thin films deposited at a flow ratio of O_2_/Ar of 0.48, the oxidation state of the Mo^0^ was not detected but an extra Mo^4+^ oxidation state with Mo 3d_5/2_ and Mo 3d_3/2_ peak positions at 229.46 eV and 232.59 eV was observed [43,46]. The oxidation states Mo^5+^ and Mo^6+^ had Mo 3d_5/2_ and Mo 3d_3/2_ peak positions at 231.50 eV and 234.63 eV and at 232.44 eV and 235.57 eV, correspondingly. The presence of Mo^5+^ and Mo^4+^ oxidation states suggested the formation of MoO_2_ and Mo_2_O_5_. Increasing the flow rate ratio to 0.56 O_2_/Ar (Figure 3e), a single oxidation state was presently associated with the Mo^6+^ oxidation state with Mo 3d_5/2_ and Mo 3d_3/2_ peak positions at 232.55 eV and 235.68 eV [45,46]. This spectrum was compatible with the presence of MoO_3_. All binding energies values for the molybdenum oxides species are displayed in Table 2.

#### 3.1.4. Raman Analyses

**Figure 4:**
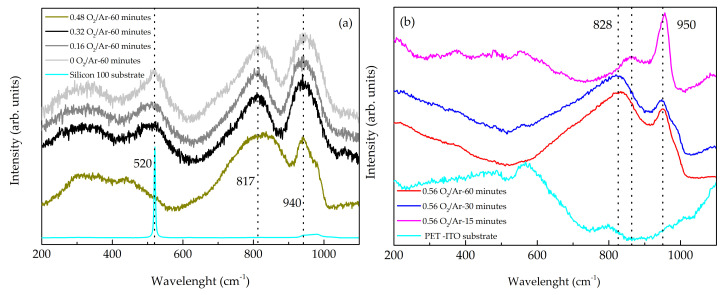
Raman spectra of the: (**a**) the first series of MoO_x_ films grown at a different flow rate ratio of O_2_/Ar; (**b**) the second series of MoO_x_ films deposited with a 0.56 flow rate ratio of O_2_/Ar and different deposition times.

The Raman spectra of the first and second series of thin films are presented in Figure 4a,b. In general, the Raman spectra of the deposited thin films are in agreement with the principle that the structure is formed by MoO_6_ octahedra, where stretching and bending vibrational modes occur in the regions 200–400 cm^−1^ and 500–1000 cm^−1^, respectively, and the narrow Raman bands detected between 920–1000 cm^−1^ are due to the vibration of terminal Mo=O bonds [47].

The Raman spectra of the samples deposited at the flow rate ratios up to 0.48 O_2_/Ar were very similar, presenting one broadband and three broad peaks. The broad featureless band observed between 200 cm^−1^ and 400 cm^−1^ could be assigned to amorphous structural nature, the first broad peak located at 520 cm^−1^ was related to the optical phonon vibrations of the silicon substrate and the bands located at 817 cm^−1^ and 940 cm^−1^, more intense and defined, could be related to the stretching vibration of doubly-connected bridge oxygen (Mo-O-Mo) groups and Mo = O vibration bond [47,48]_,_ respectively. It is important to mention that the as-deposited samples were exposed to atmospheric conditions before Raman analyses, corroborating the presence of MoO_3_ oxides as also observed by XPS. Increasing the flow rate ratio to 0.56 O_2_/Ar (Figure 4b), a shift of the 817 cm^−1^ and 940 cm^−1^ bands to higher wavenumbers was observed, indicating a predominance of the MoO_3_ base structure [49,50].

#### 3.1.5. Morphological and Thin Film Growth Features

**Figure 5:**
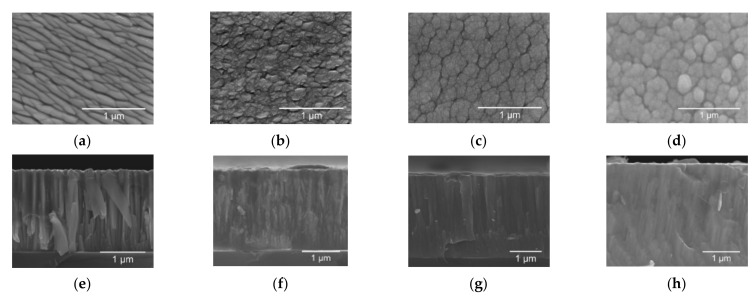
Surface SEM images of the first series of MoO_x_ films deposited on a silicon substrate with (**a**) 0 O_2_/Ar-60 min; (**b**) 0.16 O_2_/Ar-60 min; (**c**) 0.32 O_2_/Ar-60 min; (**d**) 0.48 O_2_/Ar-60 min. Cross-section SEM images of the first series of MoO_x_ films deposited on a silicon substrate with (**e**) 0 O_2_/Ar-60 min; (**f**) 0.16 O_2_/Ar-60 min; (**g**) 0.32 O_2_/Ar-60 min; (**h**) 0.48 O_2_/Ar-60 min.

**Figure 6:**
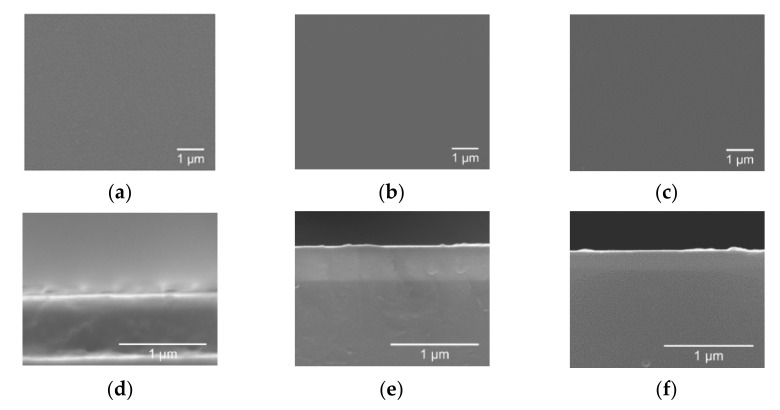
Surface SEM images of the second series of MoO_x_ films grown on a silicon substrate with (**a**) 0.56 O_2_/Ar-60 min; (**b**) 0.56 O_2_/Ar-30 min; (**c**) 0.56 O_2_/Ar-15 min. Cross-section SEM images of the second series of MoO_x_ films grown on a silicon substrate with (**d**) 0.56 O_2_/Ar-60 min; (**e**) 0.56 O_2_/Ar-30 min; (**f**) 0.56 O_2_/Ar-15 min.

**Figure 7:**
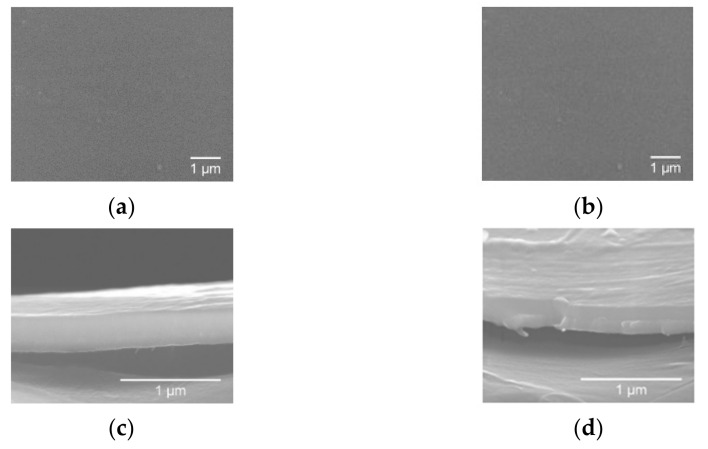
Surface SEM images of the second series of MoO_x_ films grown on polyethylene terephthalate with indium tin oxide (PET-ITO) substrate with (**a**) 0.56 O_2_/Ar-30 min; (**b**) 0.56 O_2_/Ar-15 min. Cross-section SEM images of the second series of MoO_x_ films grown on polyethylene terephthalate with indium tin oxide (PET-ITO) substrate with (**c**) 0.56 O_2_/Ar-30 min; (**d**) 0.56 O_2_/Ar-15 min.

The two-dimensional (2D) surface and cross-section morphologies of MoO_x_ thin films deposited on silicon and PET-ITO substrates have been investigated using SEM images as shown in Figure 5, Figure 6 and Figure 7.

The SEM micrographs show that Mo films grown in pure Ar atmosphere, exhibited a well-defined structure with flat elongated plate-like shaped grains in a top view and columnar grain growth in cross-section [37]. The samples deposited with a ratio of O_2_/Ar of 0.16, 0.32 and 0.48 present islands and clusters in top view that became more expressive with the increase of oxygen content. The cross-section micrographs revealed a columnar grain growth that became dense and featureless with the increase of the O_2_ flow rate.

The sample deposited at 0.56 ratio of O_2_/Ar, Figure 6, revealed a compact/dense and inexpressive morphology on both top view and cross-sections, without evidence of the presence of cracks or porosity.

In Figure 7 it is possible to observe the morphology of the thin films grown on PET-ITO substrates, which was similar to the one reported for the films deposited on silicon. Moreover, it was visible that the thickness decreased from around 910 nm (sample 0.56 O_2_/Ar-60 min) to 400 nm (sample 0.56 O_2_/Ar-30 min). Sample 0.56 O_2_/Ar-15 min showed a thickness of around 190 nm.

These film thicknesses were slightly different from the ones calculated through RBS measurements (Section 3.1.2) since those calculations depended on the material’s density in the bulk form and not of the thin film. Taking this feature into account, Table 3 shows the results of a deeper study of RBS spectra, calculating the film thickness considering different stoichiometries and calculating the film density considering the thickness estimated from the SEM measurements. The calculated density results were lower than each possible stoichiometry of molybdenum oxide, suggesting the existence of voids in the film structure. Moreover, and considering a possible statistical error of about 10 to 20% in the calculated values, the density was approximately the same for all samples indicating a similar stoichiometry. From this, it was also suggested that the calculated values were an average of a mixture of several oxidation states of molybdenum.

Table 4 shows the roughness of the samples grown at the 0.56 O_2_/Ar flow rate ratio with different deposition times by optical profilometry. Samples deposited on the PET-ITO substrate presented the lowest roughness, between 6 and 8 nm. To confirm the values of density estimated by RBS, XRR measurements were carried out for these samples without the need of using a monochromator despite the very thick layers involved. The XRR curves were characterized by a region of low incident angles for which the X-ray beam intensity was totally reflected and a region above a critical angle ($\theta_{c}$) for which the intensity fell rapidly with the incident angle with a strong dependence on the roughness. By simulating the curves, it was possible to estimate the complex refractive indexes of the layered structure, *n*. The *n* value is given by:

(1) $$n=\left(1-\delta \right)-i\beta$$

where δ parameter ranges from 10^−5^ to 10^−6^ for X-ray wavelength of approximately 1 Å, and β is related to the X-ray absorption. The critical angle $\theta_{c}$ is given by:

(2) $$\theta_{c}=\sqrt{2\delta }$$

δ in turn, is proportional to ρ through (Equation (3)):

(3) $$\delta =\frac{\frac{r_{e}\lambda^{2}}{2\pi }N_{0}\rho \sum_{i}x_{i}\left(Z_{i}+f_{i}^{’}\right)}{\sum_{i}\left(x_{i}M_{i}\right)}$$

where $r_{e}$ is the classical electron radius, $N_{o}$ is the Avogrado number, λ is the X-ray wavelength, ρ is the density, $x_{i}$ is the atomic ratio of atom i, $Z_{i}$ is the atomic number of atom i, $M_{i}$ is the atomic weight of the atom i and $f^{\prime }$ is the real part anomalous dispersion term of the atomic scattering factors for atom i. By replacing above the $f^{\prime }$ with $f^{\prime \prime }$ (imaginary part of the atomic scattering factors) the third term in the refrective index n, β, is derived (Equation (4)) [52].

(4) $$\beta =\frac{\frac{r_{e}\lambda^{2}}{2\pi }N_{0}\rho \sum_{i}x_{i}\left(Z_{i}+f_{i}^{’’}\right)}{\sum_{i}\left(x_{i}M_{i}\right)}$$

The simulations were performed using the MROX code following the dynamical theory recursive method developed by Parratt [53]. Furthermore, due to the impossibility of obtaining a reasonable fit considering homogeneous MoO_3_ layers, a function to estimate the contribution of pores using the kinematical model developed by Maaze et al. [54] was included. The contribution of the pores in the X-ray reflectivity scans was visible around 1.42° where a small shoulder suggested the small-angle X-ray scattering due to the randomly dispersed pores in the matrix. For the simulations, and to estimate the density, pores with aspherical shape and an averaged inter-distances between 10 nm and 500 nm were assumed.

**Figure 8:**
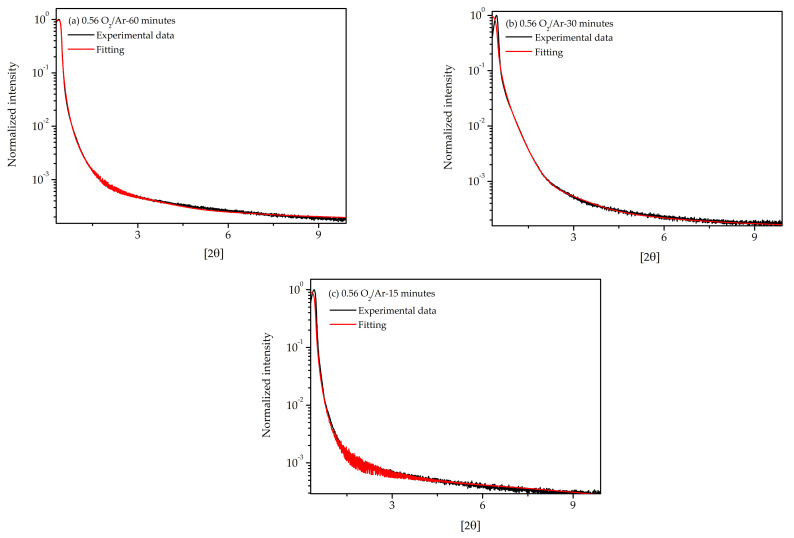
X-ray reflectivity (XRR) spectra and simulations of the film’s growth at the 0.56 O_2_/Ar flow rate during (**a**) 60, (**b**) 30 and (**c**) 15 min.

The fits were performed using a standard genetic algorithm and are presented in Figure 8. A density of around 2.4 g/cm^3^ was obtained for the surface layer, which was in the same level of the ones obtained by RBS (Table 3) and lower than the bulk density of MoO_3_ (4.69 g/cm^3^). The density derived was also an indication of an oxygen over-stoichiometry at the surface, as RBS also suggested. Although a clear improvement of the fitting was possible, considering the contribution of pores, the derived thickness in some samples did not match with the ones observed by SEM. This can be due to the limitation of this technique for the estimation of thickness when interference oscillations were not observed or it could suggest that other reasons were affecting the angular dependency of the XRR intensity at angles above the critical angle that were not considered in the fit. Small soller-slit mis-alignments or crystal heterogeneities, such as in the thickness along the high-area probed sample due to the small X-ray angle scattering events are possible reasons. However, it is out of the scope of this manuscript to deeper the outputs of the X-ray reflectivity simulation.

### 3.2. Optical Response of the Thin Films

**Figure 9:**
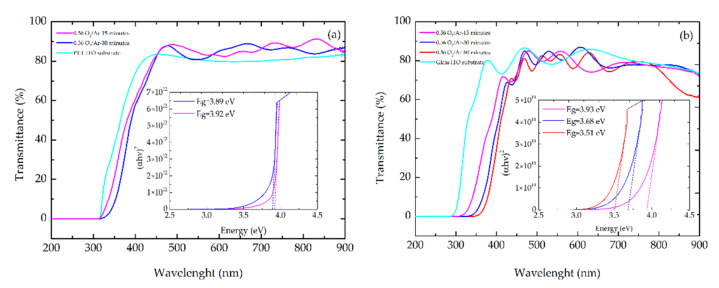
Transmission spectra and plots of ${\left(\alpha h\nu \right)}^{2}$ versus hν for direct transition in MoO_x_ films (**a**) grown on PET-ITO substrate and (**b**) grown on glass-ITO substrate at 0.56 O_2_/Ar flow rate ratio at different deposition time.

Figure 9a,b present the transmittance spectra of MoO_x_ films grown on PET-ITO and glass-ITO substrate deposited at 0.56 O_2_/Ar flow rate ratio with different deposition times. It was found that the MoO_x_ films deposited onto PET-ITO substrates exhibited higher transmittance, in the visible range, than the ones deposited onto glass-ITO substrates (maxima of 89% and 87%, respectively). The phenomenon observed in Figure 9a related to higher transmittance of MoO_x_ films deposited on PET-ITO substrate was already observed by [55,56] and it was justified by an improvement of the anti-reflective property of the ITO layer after the MoO_x_ film deposition, increasing the global transmittance. The energy band gaps for direct and indirect transitions of MoO_x_ films were obtained by the Tauc method [40], by extrapolation of the linear portion of a plot of ${\left(\alpha h\nu \right)}^{2}$ versus hν for the direct transition, and ${\left(\alpha h\nu \right)}^{\frac{1}{2}}$ versus hν for indirect transition, where α is the optical absorbance coeficiente and hν the photon energy. The maximum energy bandgap value obtained for MoO_x_ films grown on PET-ITO substrate was 3.92 eV and for MoO_x_ films grown on glass-ITO substrate was 3.93 eV. A blue shift of the bandgap value was observed with the increase of the thickness, which can be explained in terms of the extended disorder and presence of defects that could be related to the existence of substoichiometric MoO_3_ phases [40]. This direct bandgap energy behaviour could be related to a gradual increase of oxygen ion vacancies in the film with the deposition time [57].

### 3.3. Electrical Characterization

**Figure 10:**
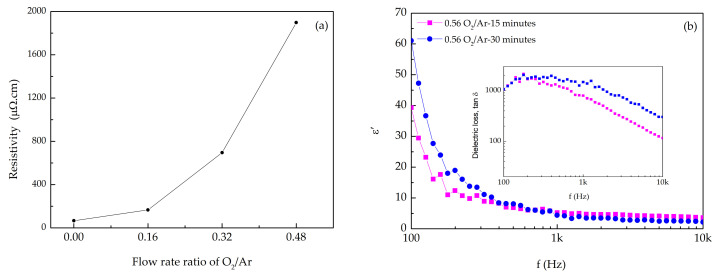
(**a**) Electrical resistivity of MoO_x_ thin films at different O_2_/Ar flow rate ratio; (**b**) Dielectric constant and loss of the samples 0.56 O_2_/Ar-30 min and 0.56 O_2_/A-15 min.

The electrical resistivity, at room temperature, of the thin films with O_2_/Ar flow ratio between 0 (oxygen absence) and 0.48 was measured using the four-probe method [58] (Figure 10a). The samples resistivity was calculated using the Equation (5):

(5) $$\rho \;=\;\frac{V}{i}wF_{3}$$

where ρ represents the resistivity, V the measured voltage, i the applied current, w the film thickness and $F_{3}$ a correction factor related to the geometry of the sample [59,60]. Correction factors were obtained through geometric series with further calculations. In the correspondent employed physical modes, the contact area was assumed infinitesimal (true in very good approximation). Geometrically, the four-point probes were collinear and equidistant. As shown in Figure 10, the electrical resistivity of the films depended on the oxygen concentration used in the sputtering atmosphere. The metallic Mo sample (Mo^0^) showed a resistivity of 65.7 µΩ.cm which was in agreement with reported studies [61]. Increasing the oxygen content, the resistivity increased to the order of hundreds of µΩ.cm. The sample prepared at 0.48 flow rate ratio of O_2_/Ar presented the highest resistivity value, in the order of thousands µΩ.cm. This sample presented a metallic-like appearance and the value of resistivity could be assigned to the presence of MoO_2_ [62]. With further increase of the oxygen concentration in the sputtered atmosphere, optical transparency was achieved, and the electrical properties changed significantly. The samples became highly resistive, a feature that was in agreement with the already results discussed.

Figure 10b presents the dielectric constant (ε’) behavior as a function of the frequency, measured by impedance spectroscopy [63], for the MoO_x_ transparent films deposited on PET-ITO substrates, for 30 and 15 min. Both samples showed similar profiles, but the sample 0.56 O_2_/Ar-15 min showed a higher ε’ in the high-frequency region. In the low-frequency region, the ε’ was high due to the Maxwell–Wagner polarization effect, i.e., due to polarization phenomenon arising from sample surface-electrode interfaces, although some possible effect of space charge due to injection electrodes, cannot be excluded. The decrease of ε’ with the increase of the frequency was expected as part of the relaxation process that should occur at very low frequency. At 1 kHz, and at room temperature, the ε’ of the sample deposited for 15 min (~5.2) was higher than the one grown for 30 min (~4.4). This difference should be related to the fact that the first one has a MoO_x_ stoichiometry with x < 3 (Figure 2). Comparing these results with the ones from the literature [64,65] that shows, in similar experimental conditions, higher dielectric constants, it is possible to claim that the differences observed might be the result of the existence of voids in the films microstructure.

### 3.4. Electrochromic Characterization

MoO_3_ thin films, with Mo^+6^ sites, are commonly colorless. The intercalation of charges into the film during the redox reaction, where Mo^+6^ ions are reduced to Mo^+5^ causes a blue coloration of the films. Further deintercalation of charges, forces the Mo^+5^ sites in the film to be reoxidized to Mo^+6^ and the film becomes to the original colorless state. Coloration and bleaching states are guided by the following electrochemical redox reaction [5,66]:

(6) $${\mathrm{MoO}}_{3}\;\left(\mathrm{colourless}\right)+{\mathrm{yLi}}^{+}+{\mathrm{ye}}^{-}↔{\mathrm{Li}}_{y}{\mathrm{MoO}}_{3}\;\left(\mathrm{dark}\right)$$

On the Li_y_MoO_3_ chemical formula Equation (1), y is named “insertion coefficient” and represents the fractional number of sites in MoO_3_ lattice able to be filled by Li^+^ ions. Usually, the final color observed with almost MoO_3_ filled is blue (dark). However, in the present work, the lattice has some MoO_x_ incorporation (as supported by XPS data) and the final reduction reaction gave a dark brown color.

**Figure 11:**
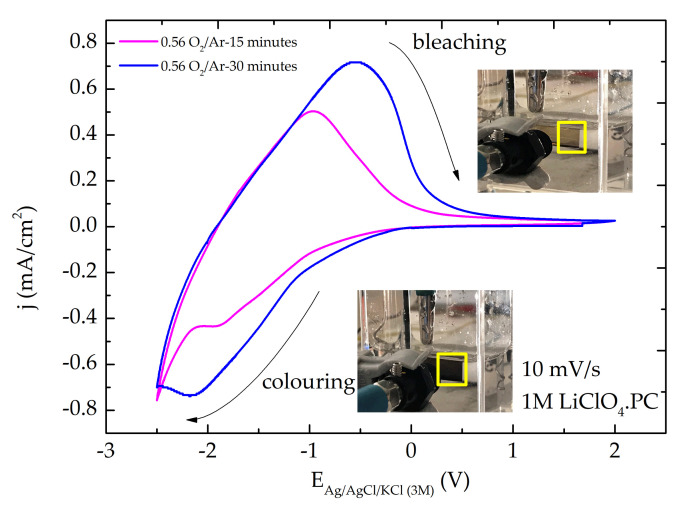
Comparison of typical CV curves of MoO_x_ thin films grown on PET-ITO substrate with different deposition times at a scan rate of 10 mV/s. The arrows indicate the scan direction.

The cyclic voltammetry experiments were conducted at the sweep rate of 10 mV/s in the 1M electrolyte solution of LiClO_4_ dissolved in propylene carbonate. It is important to mention that, for MoO_3_ films, cyclic voltammetry experiments should not be performed in aqueous solutions unless the surfaces are chemically modified to create stability [23]. Figure 11 presents typical cyclic voltammetry (CV) curves of MoO_x_ films grown on PET-ITO substrate at 0.56 flow rate ratio of O_2_/Ar and different deposition times. It was found that when the MoO_x_ films underwent a cathodic sweep (from positive to negative voltage), a continuous enhancement in the dark brown color of the film was observed. On contrary, during an anodic sweep, the film bleached and became almost colorless.

**Figure 12:**
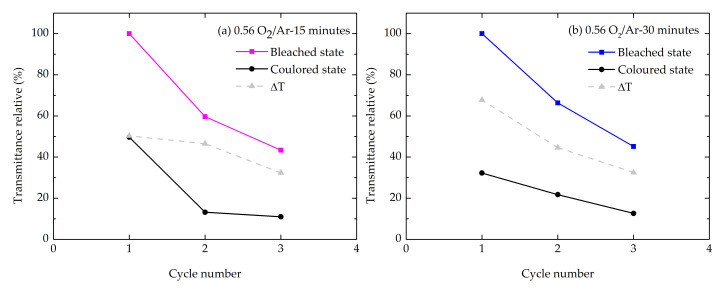
Evolution of representative transmittance of the sample (**a**) 0.56 O_2_/Ar-15 min and (**b**) 0.56 O_2_/Ar-30 min scanned at a rate of 10 mV/s. ∆T represents the relative optical transmittance difference between the colored and bleached states.

Figure 12a,b presents the evolution of relative transmittance for both samples studied. The optical transmittance decreased over time, indicating that some Li^+^ ions intercalated during the coloration cycle could not be deintercalated, resulting in the retention of a small fraction of Li^+^ ions. During the cathodic sweep, the current density increased continuously and attained a negative maximum value at the potential of −2.5 V_Ag/AgCl/KCl (3M)_ and the film exhibited a deep brown coloration when electrons and Li^+^ ions intercalated into the MoO_x_ films. Changing the potential from −2.5 to 2.0 V_Ag/AgCl/KCl (3M)_ during the anodic excursion, the current density changed from negative to positive, attaining a maximum (positive) value, decreasing after until the film was bleached. The bleaching process occurred due to the deintercalation of the electrical charges from the film structure. During the anodic scan, the film exhibitd a broad peak, whereas during cathodic scan a characteristic “spike” could be seen at the potential of −2.5 V_Ag/AgCl/KCl (3M)_ (Figure 11), in agreement with previous results [67,68]. The absence of a well-defined peak in the cathodic region was due to the back electromotive force (back emf) formed within the LiMoO_x_ (molybdenum bronze) during ion insertion [69]. In Figure 11 it is evident that the cathodic and anodic peak current densities increased upon deposition time. This fact is supported by the amount of intercalated and deintercalated Li^+^ ions determined by integration of the cathodic and anodic current, respectively, as listed in Table 5.

The film color reversibility, which correlates the intercalated/deintercalated charge, showed a higher value for the sample with a thickness of around 400 nm (deposition time of 30 min). On the other hand, the anodic current peak position in the thinner sample (~190 nm) shifted towards the lower potential inferring that the process involving a change from colored to bleached state was faster for the sample deposited with lower deposition time. The CV curve areas were found to increase with the increase of the deposition time (thinner), suggesting an increase of the intercalation capacity.

The diffusion coefficient of Li^+^ ions during the intercalation and deintercalation, into MoO_x_ host lattice, could be obtained by the Randles–Sevick relationship [29]:

(7) $$j_{p}\;=\;2.72\;x\;10^{5}n^{\frac{3}{2}}D^{\frac{1}{2}}Cv^{\frac{1}{2}}$$

where $j_{p}$ is the peak current density (A/cm^2^), n is the number of electrons involved in the redox process (assumed to be 1), D is the diffusion coefficient of Li^+^ ions (cm^2^/s), C is the concentration of active ions in the electrolyte solution (mol/cm^3^), and ν is the scan rate (V/s). Table 5 shows these different electrochromic parameters for the MoO_x_ films. It is observed that the diffusion coefficient of Li^+^ ions for both process intercalation and deintercalation was faster for the thinner sample (190 nm—deposition time of 15 min) and the variation on the mobility of the diffusing ionic species could be related with the texture of the film [68], suggesting that low roughness promoted faster inter-deintercalation processes.

Coloration efficiency $\left(\eta \right)$ is another very important electrochromic parameter that correlates the change in optical density with the charges intercalated per unit of electrode area by the following relationship [70]:

(8) $$\eta \left(\lambda \right)\;=\;\frac{\Delta OD}{\left(\frac{Q}{A}\right)}\;=\;\frac{ln\left(\frac{T_{bleached}}{T_{coloured}}\right)}{\left(\frac{Q}{A}\right)}$$

where ΔOD is the change in optical density, $\frac{T_{bleached}}{T_{coloured}}$ are the transmittances of the film in colored and bleached states, at a certain wavelength, Q is the intercalated charge, and A is the film electrode area. The maximum coloration efficiency obtained was 19.6 cm^2^/C (Table 5), observed on the MoO_x_ film grown for 15 min. Although not very high, such a value was in agreement with some previous work found in the literature [71] considering the device stability. Moreover, in the present work, the material was deposited on flexible substrates with low surface roughness.

## 4. Conclusions

Films of MoO_x_, with thickness from 190 up to 910 nm, were prepared by DC magnetron sputtering in stiff and flexible substrates.

XRD analysis shows a transition from a crystalline Mo structure to a MoOx amorphous state, occurring between 0.48 and 0.56 O_2_/Ar flow rate ratio. XPS indicates the chemistry evolution, with the increase of the O_2_/Ar ratio, from Mo^0^ up to the Mo^6+^ oxidation state. Raman corroborates these results. It was found a density value of around 2.4 g/cm^3^, supported by XRR. RBS revealed a decrease of the Mo/O ratio with the rise of O_2_/Ar flow rate ratio and time of deposition. It showed that the molybdenum profile is not homogeneous across the thickness.

The transparent and flexible films present an optical transmittance of 89%, in the visible range and an energy bandgap value of 3.92 eV. The dielectric response of these films revealed an ε’, at 300 K and 1 kHz, between 4.4 and 5.2 depending on the time of growth. The electrochromic response showed that the 0.56 O_2_/Ar-15 min film exhibits the maximum coloration efficiency (19.6 cm^2^/C), but the 0.56 O_2_/Ar-30 min film shows better reversibility.

## Figures and Tables

**Table 1:** Structural parameters of crystalline MoO_x_ films and crystallite size (nm).

Sample	Intense Peaks		Crystallite Size (nm)
	D-Spacing (Å)	(hkl)	
0 O_2_/Ar**-**60 min	2.24323	(110)	28 ± 3
	1.57868	(200)	
	1.29034	(211)	
0.16 O_2_/Ar**-**60 min	2.23039	(110)	16 ± 3
	1.58409	(200)	
	1.28323	(211)	
0.32 O_2_/Ar**-**60 min	2.42993	(111)	13 ± 2
	2.10633	(200)	
	1.48369	(220)	
	1.26528	(311)	

**Table 2:** Binding energies (eV) of the Mo 3d orbitals obtained by XPS and from literature.

Sample	Mo 3d Orbitals	Mo (0)	MoO_2_ (IV)	Mo_2_O_5_ (V)	MoO_3_ (VI)
0 O_2_/Ar- 60 min	Mo 3d_3/2_	230.50	-	234.30	235.30
	Mo 3d_5/2_	227.37	-	231.17	232.17
0.16 O_2_/Ar- 60 min	Mo 3d_3/2_	230.96	-	234.66	235.73
	Mo 3d_5/2_	227.83	-	231.53	232.60
0.32 O_2_/Ar- 60 min	Mo 3d_3/2_	230.96	-	234.53	235.52
	Mo 3d_5/2_	228.71	-	231.40	232.37
0.48 O_2_/Ar- 60 min	Mo 3d_3/2_	-	232.59	234.63	235.57
	Mo 3d_5/2_	-	229.46	231.50	232.44
0.56 O_2_/Ar- 60 min	Mo 3d_3/2_	-	-	-	235.67
	Mo 3d_5/2_	-	-	-	232.56
From literature [41,42,44]	Mo 3d_3/2_	230.85	232.6	234.62	235.85
	Mo 3d_5/2_	227.70	229.4	231.63	232.65

**Table 3:** Samples thickness calculated using RBS fit results and considering different molybdenum oxides and density estimation considering the thickness measured by SEM.

Sample	Areal Density	Thickness (nm)		^+^ Density (g/cm^3^)
	(10^15^ atm/cm^2^)	*	**	
0.48 O_2_/Ar-60 min	429,949	-	-	-
0.56 O_2_/Ar-60 min	4900	385	531	2.74
0.56 O_2_/Ar-30 min	1658	122	166	1.97
0.56 O_2_/Ar-15 min	862	61.4	84.7	2.09

* considering the MoO_2_ density 6.47 g/cm^3^ [51]; **considering the MoO_3_ density 4.69 g/cm^3^ [16]; ^+^ Density estimated considering the stoichiometry obtained from fit of RBS data and the thickness estimated from the SEM measurements.

**Table 4:** Surface roughness (root mean square height, Sq) measured by optical profilometry of the samples grown at flow rate of 0.56 O_2_/Ar with different deposition times.

Sample	Substrate	Sq (nm)
0.56 O_2_/Ar-15 min	PET-ITO	8
0.56 O_2_/Ar-30 min		6
0.56 O_2_/Ar-15 min	Glass-ITO	11
0.56 O_2_/Ar-30 min		13
0.56 O_2_/Ar-60 min		42

**Table 5:** Cyclic voltammetry measurements of the 0.56 O_2_/Ar films growth at 15 and 30 min.

Sample		0.56 O_2_/Ar-15 min	0.56 O_2_/Ar-30 min
Scan rate (mV/s)		10	10
Cathodic current density, j_pc_ (A/cm^2^)		7.57 × 10^−4^	6.83 × 10^−4^
Cathodic peak potential, V_pc_ (V_Ag/AgCl/KCl (3M)_)		−2.50	−2.46
Anodic current density, j_pa_ (A/cm^2^)		5.01 × 10^−4^	6.56 × 10^−4^
Anodic peak potential, V_pa_ (V_Ag/AgCl/KCl (3M)_)		−1.00	−0.68
Diffusion coefficient (cm^2^s^−1^)	D_insertion_	7.74 × 10^−10^	6.31 × 10^−10^
	D_extraction_	3.39 × 10^−10^	5.81 × 10^−10^
Intercalated charge (C/cm^2^)		0.077	0.109
Deintercalated charge (C/cm^2^)		0.067	0.099
Reversibility (%)		87%	91%
Coloration efficiency at 630 nm (cm^2^/C)		19.6	10.2

## References

[1] Yu X., Marks T.J., Facchetti A. (2016). Metal oxides for optoelectronic applications. Nat. Mater..

[2] Lorenz M., Rao M.S.R., Venkatesan T., Fortunato E., Barquinha P., Branquinho R., Salgueiro D., Martins R., Carlos E., Liu A. (2016). The 2016 oxide electronic materials and oxide interfaces roadmap. J. Phys. D Appl. Phys..

[3] Ashrit P. (2017). Transition Metal Oxide Thin Film-Based Chromogenics and Devices.

[4] Monk P., Mortimer R., Rosseinsky D. (2007). Electrochromism and Electrochromic Devices.

[5] Granqvist C.G. (1995). Handbook of Inorganic Electrochromic Materials.

[6] Granqvist C.G. (2012). Oxide electrochromics: An introduction to devices and materials. Sol. Energy Mater. Sol. Cells.

[7] Arvizu M.A., Granqvist C.G. (2016). Gunnar A Niklasson, Rejuvenation of degraded electrochromic MoO3 thin films made by DC magnetron sputtering: Preliminary results. J. Phys..

[8] Miyata N., Akiyoshi S. (1985). Preparation and electrochromic properties of rf-sputtered molybdenum oxide films. J. Appl. Phys..

[9] Boufker K. (1995). Lithiation study of molybdenum oxide thin films: Application to an electrochromic system. J. Appl. Electrochem..

[10] Nirupama V., Sekhar M.C., Subramanyam T.K., Uthanna S. (2010). Structural and electrical characterization of magnetron sputtered MoO3 thin films. J. Phys. Conf. Ser..

[11] Senthilkumar R., Anandhababu G., Mahalingam T., Ravi G. (2016). Photoelectrochemical study of MoO3 assorted morphology films formed by thermal evaporation. J. Energy Chem..

[12] Lin C.-Y., Wang C.-M., Kao K.-S., Chen Y.-C., Liu C.-C. (2010). Electrochromic properties of MoO3 thin films derived by a sol-gel process. J. Sol.-Gel. Sci. Technol..

[13] Chang C.-C., Chi P.-W., Chandan P., Lin C.-K. (2019). Electrochemistry and Rapid Electrochromism Control of MoO_3_/V_2_O_5_ Hybrid Nanobilayers. Materials.

[14] Sivakumar R., Gopinath C.S., Jayachandran M., Sanjeeviraja C. (2007). An electrochromic device (ECD) cell characterization on electron beam evaporated MoO3 films by intercalating/deintercalating the H+ ions. Curr. Appl. Phys..

[15] Donnadieu A., Davazoglou D., Abdellaoui A., Bataillon P.E. (1988). Structure, Optical and Electro-optical prpoerties of polycrystalline WO3 and MoO3 thin films prepared by chemical vapour deposition. Thin Solid Films.

[16] Lee Y.J., Nichols W.T., Kim D., Kim Y.D. (2009). Chemical vapour transport synthesis and optical characterization of MoO_3_ thin films. J. Appl. Phys..

[17] Chang C.-C., Luo J.-Y., Chen T.-K., Yeh K.-W., Huang T.-W., Hsu C.-H., Chao W.-H., Ke C.-T., Hsu P.-C., Wang M.-J. (2010). Pulsed laser deposition of (MoO_3_)1 - X(V_2_O_5_)x thin films: Preparation, characterization and gasochromic studies. Thin Solid Films.

[18] Ramana C.V., Hussain O.M., Julien C.M. (2006). Electronic Properties and Performance upon Lithium Intercalation of MoO_3_ Thin Grown by PLD. ECS-Electrochem. Soc..

[19] Bouzidi A., Benramdane N., Tabet-Derraz H., Mathieu C., Khelifa B., Desfeux R. (2003). Effect of substrate temperature on the structural and optical properties of MoO_3_ thin films prepared by spray pyrolysis technique. Mater. Sci. Eng. B Solid-State Mater. Adv. Technol..

[20] Afify H.H., Hassan S.A., Abouelsayed A., Demian S.E., Zayed H.A. (2016). Synthesis, characterization and structural control of nano crystalline molybdenum oxide MoO3 single phase by low cost technique. Mater. Chem. Phys..

[21] Dai T., Ren Y., Qian L., Liu X. (2018). Characterization of Molybdenum Oxide Thin Films Grown by Atomic Layer Deposition. J. Electron. Mater..

[22] Vos M.F.J., Macco B.B., Thissen N.F.W., Bol A.A., Kessels W.M.M. (2016). Atomic layer deposition of molybdenum oxide from (NtBu)2(NMe_2_)2Mo and O_2_ plasma. J. Vac. Sci. Technol. A Vac. Surf. Film..

[23] Yao J.N., Loo B.H., Fujishima A. (1990). A Study of the Photochromic and Electrochromic Properties of MoO3 Thin Films. Phys. Chem..

[24] Granqvist C.G. (2016). Electrochromics and Thermochromics: Towards a New Paradigm for Energy Efficient Buildings. Mater. Today Proc..

[25] Hosono H. (2007). Recent progress in transparent oxide semiconductors: Materials and device application. Thin Solid Films.

[26] Granqvist C.G. (2008). Oxide electrochromics: Why, how, and whither. Sol. Energy Mater. Sol. Cells.

[27] Wen R.T., Niklasson G.A., Granqvist C.G. (2015). Strongly improved electrochemical cycling durability by adding iridium to electrochromic nickel oxide films. ACS Appl. Mater. Interfaces.

[28] Arvizu M.A., Triana C.A., Stefanov B.I., Granqvist C.G., Niklasson G.A. (2014). Electrochromism in sputter-deposited W-Ti oxide films: Durability enhancement due to Ti. Sol. Energy Mater. Sol. Cells.

[29] Usha N., Sivakumar R., Sanjeeviraja C. (2015). Electrochromic properties of radio frequency magnetron sputter deposited mixed Nb_2_O_5_:MoO_3_ (95:5) thin films cycled in H+ and Li+ ions. Mater. Sci. Semicond. Process..

[30] Usha N., Sivakumar R., Sanjeeviraja C., Balasubramaniam R., Kuroki Y. (2016). Mixed Nb_2_O_5_:MoO_3_(95:5 and 85:15) thin films and their properties for electrochromic device applications. J. Mater. Sci. Mater. Electron..

[31] Granqvist C.G. (2016). Recent progress in thermochromics and electrochromics: A brief survey. Thin Solid Films.

[32] Domingues R.P., Rodrigues M.S., Lopes C., Pedrosa P., Alves E., Barradas N.P., Borges J., Vaz F. (2019). Thin films composed of metal nanoparticles (Au, Ag, Cu) dispersed in AlN: The influence of composition and thermal annealing on the structure and plasmonic response. Thin Solid Films.

[33] Barradas N.P., Jeynes C., Webb R.P. (2017). Simulated annealing analysis of Rutherford backscattering data. Appl. Phys. Lett..

[34] Pethe S.A., Takahashi E., Kaul A., Dhere N.G. (2012). Effect of sputtering process parameters on film properties of molybdenum back contact. Sol. Energy Mater. Sol. Cells.

[35] Kashyout A.E.B., Soliman H.M.A., Abou H., Aly P., Fathy M. (2011). Preparation and characterization of DC sputtered molybdenum thin films. Alex. Eng. J..

[36] Pachlhofer J.M., Martín-Luengo A.T., Franz R., Franzke E., Köstenbauer H., Winkler J., Bonanni A., Mitterer C. (2017). Industrial-scale sputter deposition of molybdenum oxide thin films: Microstructure evolution and properties. J. Vac. Sci. Technol. A Vac. Surf. Film..

[37] Pachlhofer J.M., Jachs C., Franz R., Franzke E., Mitterer C. (2016). Structure evolution in reactively sputtered molybdenum oxide thin fi lms. Vaccum.

[38] Arvizu M.A., Tomás S.A. (2017). Influence of Thermal Annealings in Argon on the Structural and Thermochromic Properties of MoO 3. Int. J. Thermophys..

[39] Bhatia S., Khanna A. (2015). Structural and optical properties of molybdenum trioxide thin films. AIP Conf. Proc..

[40] Ponce-Mosso M., Pérez-González M., García-Tinoco P.E., Crotte-Ledesma H., Morales-Luna M., Tomás S.A. (2018). Enhanced photocatalytic activity of amorphous MoO3 thin films deposited by rf reactive magnetron sputtering. Catal. Today.

[41] Shi Y., Guo B., Corr S.A., Shi Q., Hu Y.-S., Heier K.R., Chen L., Seshadri R., Stucky G.D. (2009). Ordered mesoporous metallic MoO_2_ materials with highly reversible lithium storage capacity. Nano Lett..

[42] Wagner C.D., Riggs W.M., Davis L.E., Moulder J.F., Muilenberg G.E. (1979). Handbook of X-Ray Photoelectron Spectroscopy.

[43] Fleisch T.H. (1982). An XPS study of the UV reduction and photochromism of MoO3 and WO3. J. Chem. Phys..

[44] Murphy N.R., Sun L., Grant J.T., Jones J.G., Jakubiak R. (2015). Molybdenum Oxides Deposited by Modulated Pulse Power Magnetron Sputtering: Stoichiometry as a Function of Process Parameters. J. Electron. Mater..

[45] Morales-Luna M., Tomás S.A., Arvizu M.A., Pérez-González M., Campos-Gonzalez E. (2017). The evolution of the Mo5+ oxidation state in the thermochromic effect of MoO_3_ thin films deposited by rf magnetron sputtering. J. Alloy. Compd..

[46] Choi J.G., Thompson L.T. (1996). XPS study of as-prepared and reduced molybdenum oxides. Appl. Surf. Sci..

[47] Gesheva K.A., Ivanova T. (2006). A low-temperature atmospheric pressure CVD process for growing thin films of MoO3 and MoO3-WO3 for electrochromic device applications. Chem. Vap. Depos..

[48] Camacho-lópez M.A., Escobar-alarcón L., Picquart M., Arroyo R., Córdoba G., Haro-poniatowski E. (2011). Micro-Raman study of m-MoO_2_ to α-MoO_3_ transformation induced by cw-laser irradiation. Opt. Mater..

[49] Lee S.-H., Cheong H.M., Tracy C.E., Mascaranhas A., Benson D.K., Deb S.K. (1999). Raman spectroscopic studies of electrochromic a-MO3. Electrochim. Acta.

[50] Lee S., Seong M.J., Tracy C.E., Mascarenhas A., Pitts J.R., Deb S.K. (2002). Raman spectroscopic studies of electrochromic a-MoO_3_ thin films. Solid State Ionics.

[51] Pergament A.L., Malinenko V.P., Aleshina L.A., Kazakova E.L., Kuldin N.A. (2014). Electrical Switching in Thin Film Structures Based on Transition Metal Oxides. J. Exp. Phys..

[52] Yasaka M. (2010). X-ray thin-film measurement techniques. Rigaku J..

[53] Parratt L.G. (1954). Surface studies of solids by total reflection of X-rays. Phys. Rev..

[54] Mâaza M., Gibaud A., Sella C., Pardo B., Dunsteter F., Corno J., Bridou F., Vignaud G., Désert A., Menelle A. (1999). X-ray scattering by nano-particles within granular thin films, investigation by grazing angle X-ray reflectometry. Eur. Phys. J. B.

[55] Pat S., Özmumcu M., Ekem N., Özkan M., Korkmaz Ş., Balbaǧ M.Z. (2010). Antireflective coating on polyethylene terephthalate by thermionic vacuum arc. J. Plast. Film Sheeting.

[56] Ko Y.H., Kim M.S., Yu J.S. (2012). Controllable electrochemical synthesis of ZnO nanorod arrays on flexible ITO/PET substrate and their structural and optical properties. Appl. Surf. Sci..

[57] Arasu P.A., Williams R.V. (2015). Effect of annealing temperature on structural and optical parameters of sol-gel routed molybdenum oxide thin film. Surf. Rev. Lett..

[58] Valdes L.B. (1954). Resistivity Measurements on Germanium for Transistors. Proc. IRE.

[59] Borges J.N.P., Martin N., Barradas N., Alves E., Eyidi D., Beaufort M., Riviere J., Vaz F., Marques L.S.A. (2012). Electrical properties of AlNxOy thin films prepared by reactive magnetron sputtering. Thin Solid Films.

[60] Smits F.M. (1958). Measurement of Sheet Resistivities with the Four-Point Probe. Bell Syst. Tech. J..

[61] Dai X., Zhou A., Feng L., Wang Y., Xu J., Li J. (2014). Molybdenum thin films with low resistivity and superior adhesion deposited by radio-frequency magnetron sputtering at elevated temperature. Thin Solid Films.

[62] Oh M.S., Yang B.S., Lee J.H., Oh S.H., Lee U.S., Kim Y.J., Kim H.J., Huh M.S. (2012). Improvement of electrical and optical properties of molybdenum oxide thin films by ultralow pressure sputtering method. J. Vac. Sci. Technol. A Vac. Surf. Film..

[63] Fernandes P.A., Salomé P.M.P., Graça M.P.F. (2016). Electrical Measurements: Introduction, Concepts and Applications.

[64] Saad E.A.I. (2005). Dielectric properties of molybdenum oxide thin films. J. Optoelectron. Adv. Mater..

[65] Parmendu Kant K., Srivastava R. (1975). Dielectric Permitiviy and Breakdown Strength of Molybdenum Trioxide Films. J. Phys. Soc. Jpn..

[66] Bélanger D., Laperrière G. (1990). Electrochromic Molybdenum Trioxide Thin Film Preparation and Characterization. Chem. Mater..

[67] Patil P.R., Pawar S.H., Patil P.S. (2000). Electrochromic properties of tungsten oxide thin films deposited by solution thermolysis. Solid State Ionics.

[68] Faughnan B.W., Crandall R.S. (1977). Optical properties of mixed-oxide WO3/MoO3 electrochromic films Cite. Appl. Phys. Lett..

[69] Monk P.M.S., Ali T., Partridge R.D. (1995). The effect of doping electrochromic molybdenum oxide with other metal oxides: Correlation of optical and kinetic properties. Solid State Ionics.

[70] Hsu C.-S., Chan C.-C., Huang H.-T., Peng C.-H., Hsu W. (2008). Electrochromic properties of nanocrystalline MoO 3 thin films. Thin Solid Films.

[71] Turel O., Hacioglu S.O., Coskun S., Toppare L., Unalan H.E. (2017). Sequential Deposition of Electrochromic MoO_3_ Thin Films with High Coloration Efficiency and Stability. J. Electrochem. Soc..

